# Effective instructional strategies and technology use in blended learning: A case study

**DOI:** 10.1007/s10639-021-10544-w

**Published:** 2021-06-08

**Authors:** Meina Zhu, Sarah Berri, Ke Zhang

**Affiliations:** Learning Design and Technology, Wayne State University, Detroit, MI USA

**Keywords:** Instructional strategies, Learning technology, Blended learning, Blended courses

## Abstract

This case study explored effective instructional strategies and technology use in blended learning (BL) in a graduate course in the USA. Varied forms of data were collected, including (1) semi-structured interviews with students, (2) mid-term and final course evaluations, (3) two rounds of online debates, (4) four weeks of online reflection journals, and (5) the instructor’s reflections. Thematical analysis and descriptive statistics were conducted to analyze qualitative and quantitative data respectively. Multiple methods were employed to establish trustworthiness of the study. Effective and ineffective instructional strategies and technology uses were identified in BL. The findings indicated that students valued real-time interactions with peers and the instructor. However, inappropriate asynchronous discussions were considered less effective in BL. In addition, immediate feedback from peers and the instructor motivated learners and improved the quality of their work. Learning technologies played a critical role in BL, but the use of learning technologies should be simplified and streamlined. Technical support was essential to reduce learners’ cognitive load.

## Introduction

An increasing number of universities are adopting blended learning (BL) (Porter et al., 2014), which combines face-to-face (F2F) instruction with online instruction (Bonk & Graham, 2006; Voos, 2003). As a paradigm shift from teaching to learning (Nunan et al., 2000), researchers predicted that BL has the potential to be widely adopted in higher education (Norberg et al., 2011) and to transform F2F learning (Donnelly, 2010). Currently, due to the COVID-19 pandemic, BL is likely to increase in various forms around the world (Kim, 2020).

Research has found a range of positive effects of BL (Means et al., 2013; Vella et al., 2016), such as engaging learners (Henrie et al., 2015; Owston et al., 2013), reducing dropout rates (López-Pérez et al., 2011), enhancing knowledge construction and problem-solving abilities (Bridges et al., 2015), improving performance (Deschacht & Goeman, 2015; Shea & Bidjerano, 2014), increasing attendance and satisfaction (Stockwell et al., 2015), and providing a strong sense of community in the learning experiences (Rovai & Jordan, 2004). Researchers have also found that effective blended course design and instruction could make students feel connected with others (e.g., Cocquyt et al., 2017). In addition to the benefits of BL, some research focused on the affordance of the emerging technologies for collaborative learning (Gan et al., 2015). However, studies also show that technologies could hinder teaching and learning if not used properly (Bower et al., 2015; Park & Bonk, 2007). Understanding the specific strategies and technology use will be critical to the design of effective BL.

Despite its proven and potential advantages, more research on effective instructional strategies in BL is imperative. This study investigated both instructional strategies and technology uses in BL in a graduate course in the USA. It intended to inform researchers and practitioners of effective instructional strategies in BL and to help leverage learning technologies in blended courses.

## Literature review

### Blended learning

BL is a type of delivery mode that integrates both F2F and online instructions (Bonk & Graham, 2006; Graham, 2004). Online instruction through synchronous or asynchronous communications is supported with emerging learning technologies (Norberg et al., 2011), so, to succeed in such an environment, students must be comfortable using the technologies (Holley & Oliver, 2010; Song et al., 2004). However, not all students are familiar with all learning technologies, thus technical support is essential in BL (Graham, 2004; Johnson, 2017). In addition, scholars (e.g., Lee et al., 2011) believe that effective technical support can positively influence students’ learning satisfaction. Students who are more familiar with learning technologies are also found more active in learning activities (Min, 2010).

Research has identified diverse advantages of BL. For example, Graham and colleagues identified three primary benefits of BL: (1) enhancing pedagogy, (2) increasing access and flexibility, and (3) improving cost-efficiency (Graham et al., 2005). Research indicates that BL increases learning effectiveness, efficiency, satisfaction (Garrison & Kanuka, 2004; Graham, 2013; Martínez-Caro & Campuzano-Bolarin, 2011), performance (Boyle et al., 2003; Lim & Morris, 2009), engagement (Owston et al., 2013), attendance (Stockwell et al., 2015), and fosters collaboration (So & Brush, 2008). For instance, research conducted at the University of Central Florida has found that the success rates for BL were higher than either fully traditional face-to-face or completely online courses (Graham, 2013). Some studies also suggest that BL improved students’ knowledge construction and problem-solving skills (Bridges et al., 2015). Second, BL has great potentials to increase access and flexibility (Graham, 2006; Moskal et al., 2013). With flexibility in time and location for learning (King & Arnold, 2012), BL potentially increases access to education (Shea, 2007). Last, BL may improve the cost-effectiveness of education (Graham, 2013), as it reduces operational costs compared to traditional on-campus learning (Vaughan, 2007; Woltering et al., 2009).

### Interactions and discussions in BL

Learning interactions are essential for knowledge acquisition and skill development (Barker, 1994). Moore (1989) categorized three types of interactions, those amongst learners, between learner and instructor, and learner-content interactions. Learner-learner interactions may stimulate two-way collaborative learning (Kuo & Belland, 2016; Moore, 1989), and they are particularly important in online courses (Anderson, 2008). Learner-instructor interactions streamline communications between learner and instructor and also facilitates the delivery of instructions, guidance, and support to learners (Kuo & Belland, 2016; Moore, 1989). Learner-content interaction is typically one-way communication, in which learners interact with the content (Kuo & Belland, 2016; Moore, 1989), although emerging technologies have made it possible to dynamically generate highly customized content or learning experiences. Students can interact with peers, instructors, and learning content in BL (Moore, 1989), especially through interactive technologies (Anderson, 2008) and appropriate pedagogies.

One of the strengths of BL is that both online and face-to-face (F2F) sessions may facilitate ample opportunities for interactions (Fryer & Bovee, 2018; Johnson, 2017). Evidently, interactions are positively related to learning outcomes and learners’ satisfaction in BL (Kang & Im, 2013; Kuo & Belland, 2016; Kurucay & Inan, 2017; Wei et al., 2015). Particularly, learner-learner interaction is important for their achievement and sense of belongings (Bernard et al., 2009; Diep et al., 2017), which strengthens a sense of community (Lidstone & Shield, 2010).

Successful discussions serve as a powerful instructional strategy in higher education (Ellis & Goodyear, 2010; Rovai, 2007), as peer discussions encourage students to construct knowledge through active communications (Hamann et al., 2012; Huerta, 2007; Vonderwell, 2003). While learners demanding emotional or cognitive support would require timely and considerate facilitations from the instructor in BL (Butz et al., 2014; Szeto & Cheng, 2016). Discussions in BL could happen in either F2F or online sessions or continue in both settings. In F2F discussions, learners could share ideas and receive an immediate response; whereas, online discussions added advantages that could not be achieved in F2F settings (Joubert & Wishart, 2012; Richardson & Ice, 2010). For example, in online asynchronous discussions, students have more time to read, digest, and reflect upon learning materials, which promotes critical thinking (Putman et al., 2012; Williams & Lahman, 2011). However, online discussions, if without sufficient support or stimulus, can be superficial or wordy yet without depth (Angeli et al., 2003; Wallace, 2003). Therefore, researchers have proposed different strategies to organize discussions in F2F or online instructions (Guiller et al., 2008). Darabi and Jin (2013), for example, suggested sharing example posts with students for discussions. However, how to increase interaction and promote effective discussion is still an open question.

### Online debate

Structured debates may increase motivation, enhance critical thinking, and trigger affective communication skills in learners (Alen et al., 2015; Howell & Brembeck, 1952; Jagger, 2013; Liberman et al., 2000; Zare & Othman, 2015). Participating in debates can enhance learners’ confidence, and promote substantive knowledge and practice skills (Alen et al., 2015; Blackmer et al., 2014; Doody & Condon, 2012; Keller et al., 2001). With time flexibility, online debates also allow extended reflections and autonomy (Mutiaraningrum & Cahyono, 2015; Park et al., 2011; Weeks, 2013). Despite the rich literature on F2F debates, few research is available on online debates for learning (Mitchel, 2019). Thus, exploring how to leverage debate in online environment is imperative.

### Feedback in BL

Through learner-learner interactions, learners support each other on academic and non-academic issues (Lee et al., 2011). Research indicates that peer interactions and support correlate positively with learning outcomes (Ashwin, 2003; Chu & Chu, 2010; Lee et al., 2011). Peer feedback, a type of learner-learner interaction, invites learners to review their peers’ drafts or work in progress and provides feedback for improvement (Topping et al., 2000). It further encourages learners to make revisions or modifications according to peer feedback (Li et al., 2010; Zhang & Toker, 2011). Formative peer-feedback encourages learners to focus on learning rather than grades (Fluckiger et al., 2010). Thus, it enhances the quality of students’ work (Aghaee & Keller, 2016) and helps to build a learning community together (Rovai, 2002). It is also effective in enhancing knowledge acquisition as found in computer science courses (Venables & Summit, 2003). Reportedly, learners value the experience of reviewing others’ work and learning from fellow students (Li et al., 2010). Immediate feedback is critical in motivating learners (Denton et al., 2008) and increasing learners’ satisfaction (Lee et al., 2011). Otherwise, when feedback is delayed or not available, learners may lose motivation (Higgins et al., 2002) and miss opportunities to seek alternative strategies for solutions (Earley et al., 1990; Stein & Wanstreet, 2008). Therefore, it is vital to explore feedback strategies from both instructor and peers in BL.

The purpose of this study is to investigate effective, as well as ineffective instructional strategies and technology usage in a blended course. The following three research questions guided this study:

1. In BL, what instructional strategies are more effective or ineffective, and why?
2. From graduate students’ perspective, what are the advantages and disadvantages of BL?
3. How can BL leverage learning technologies?

## Research design

A case study can provide an in-depth and detailed examination of the situation and its contextual conditions (Thomas, 2011; Yin, 1994). Thus, a mixed-method single case study was designed to explore learners’ experiences in BL within the authentic context.

### Context and participants

The study was conducted in a graduate-level blended course at a public university in the Midwest of the USA in the Fall semester of 2019. This course introduced the foundations of instructional technology. Participants were volunteers recruited from the class, including both male (n = 2) and female (n = 4). Five participants were advanced Ph.D. students, and one was in a certificate program, and they were all majoring in learning design and technology. Participants were non-traditional students with a full-/part- time job while enrolled in the course. All participants had professional experiences as teachers, trainers, or instructional designers. Five of the six participants were native English speakers.

The learning activities in this BL course included paper analyses, online reading reflection journals or discussions, online debates, annotated bibliographies, presentations, and a final exam. The blended course included seven bi-weekly F2F sessions and eight weeks of online sessions. All F2F class meetings were arranged in the evening to accommodate students’ working schedules. In four out of eight online sessions, students were required to read weekly articles related to the topics and to post reading reflection journals in Canvas, a course learning management system (LMS). They were also encouraged to comment on peers’ reading reflection journals.

In addition, between Week 2 and 6, students participated in two rounds of online debates via Nuclino, a free online team collaborative tool with graduate students in a similar course at another university. Five groups of four were formed from the two classes, with two students from each university per group. Students conducted online debates within each group. The two students with the Authors’ role summarized the chapter from the authors’ perspective as well as presented their personal thoughts. Then, the two students from the other university followed the same procedure from the Responders’ perspective. Last, the two students with the Author’s role rejoined the conversation. Only discourses of the consenting participants were analyzed for this study. On Zoom, students also had the opportunity to meet with five guest speakers, who were either the authors of the textbooks or the authors of the readings assigned in the course. In general, each talk lasted 40–50 min, including 10–15 min for questions and answers.

To provide detailed feedback on students’ online discussions and assignments, the instructor used One Drive, an online collaboration tool adopted by the university. In One Drive, the instructor created a folder for each student and uploaded the feedback on the assignments into each folder. In F2F sessions, the instructor summarized students’ achievements and progress from the previous week and then presented new content. To create a personalized learning environment, the instructor used individual student’s names and provided examples that are highly related to students’ education and job backgrounds. After that, students presented their paper analysis, and the rest of the class provided feedback according to the same rubric. To ensure openness and effectiveness of peer feedback, the instructor highlighted that the peer evaluation scores would not be counted toward the final grade and constructive peer feedback was more important than just polite compliments.

### Data sources

Data sources in this study included: (1) semi-structured interviews with six students, (2) mid-term and final course evaluations from seven students, (3) two rounds of online debates, (4) four weeks of online reading reflection journals, and (5) the instructor’s reflections. Six semi-structured interviews were conducted at the end of the semester via Zoom. The interview protocol included eight questions, such as background information of interviewees, the activities that students thought effective or ineffective, the role of technologies, and suggestions on technology use in BL. Each interview lasted approximately 30 min and was audio-recorded (Table 1).

**Table 1:** Interviewee’s profile

Pseudo Name	Gender	Prior BL experience	Teaching/design experience in BL	Professional background
Cindy	F	Y	N	High school math teacher for six years
Lana	F	Y	Y	Full-time Graduate student & research assistant at a research one university with professional experiences in nonprofit program development and training
Linda	F	Y	Y	Instructional designer at a School of Medicine in higher education
Sam	M	Y	Y	Training manager at an automotive company for over 28 years
Shawn	M	N	N	High school teacher for 16 years and a media company owner
Zoe	F	Y	N	Full-time Graduate student & research assistant at a research one university with 5 years of professional experiences in IT and HR

The two rounds of online debates of five groups generated 30 discussion posts in total. Each group generated six discussion posts with three posts per round. The first-round of online debate generated 9,397 words, and the second round generated 7,283 words in total.

The online reading reflection journals and comments were posted on Canvas. In each week, students addressed three questions: (1) What are the important ideas in the readings? (2) Why are they important to you? And (3) what are the implications for your research or practice? Students were asked to post their original post, a reading reflection journal, to earn points. They were also encouraged to comment on peers’ posts, while such comments were not graded.

All students anonymously completed a mid-term course evaluation and a final course evaluation during F2F sessions without the instructor’s presence. Qualitative data were collected from the course evaluations and analyzed for this study.

### Data analyses

All qualitative data were analyzed by two researchers to ensure trustworthiness. The researchers used thematical analysis (Braun et al., 2014) to analyze the interviews, online debate and discussion, and the qualitative data from the course evaluations. The interviews were transcribed by the researchers, and first-level member checking was conducted with the interviewees. Once the researchers transcribed the data verbatim (Paulus et al., 2013) and confirmed the transcripts, thematic analysis was conducted. One of the researchers read the interview transcripts and suggested initial codes, categories, and themes, and two of the researchers then discussed them, resolved any disagreements, and finalized coding. The same protocol was followed to analyze online debate discourse and reflection journals.

## Findings

### Research question 1 (RQ1)

In BL, what instructional strategies are more effective or ineffective, and why?

#### Effective strategies

**Fig. 1:**
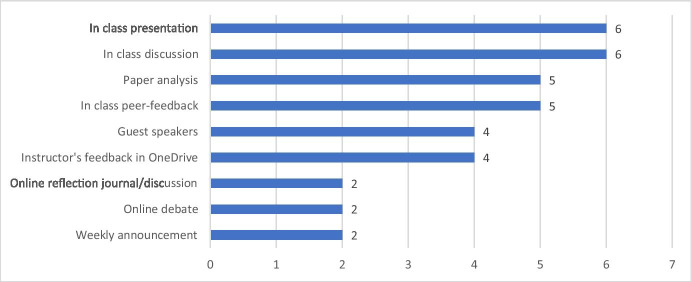
Learning activities that students perceived as effective

Students had positive perceptions of the effectiveness of learning in the blended course. In the mid-term evaluation, regarding item “overall, this course has provided an effective learning experience,” students rated 4.3/5. The specific activities favored by students were in-class presentations, discussions, peer-feedback, and paper analyses. Figure 1 shows the activities valued by students from the mid-term evaluation. Regarding the reasons why they thought F2F discussions and presentations effective, they expressed that these activities helped construct knowledge and build a learning community. For instance, in the interview, Lana said, “I would say that the presentations were really helpful for me both planning a presentation and watching my colleagues’ presentations.” Shawn shared a similar opinion in the interview “I would say that to be quite honest, the most effective thing that we did was just talking in class… I really enjoyed the conversation. I felt like because we were a smaller group… But I really acquired knowledge and really tried to listen to what people had to say.” In addition, in the course evaluation, one student mentioned: “I enjoy an interactive class atmosphere that allows us to test ideas and explain our understanding from our different perspectives.” The final course evaluation also supported that students had a positive attitude towards group interaction (M = 4.9). As the instructor of the course, the first author also noticed that students actively participated in the classroom discussion and shared their teaching or instructional design experiences and opinions with each other.

Students also perceived peer feedback in F2F sessions as effective. In the course evaluation, a student commented: “I enjoy hearing from my colleagues in the class. I feel like everyone in the class practices this in their daily lives, therefore, I value their feedback. I get more out of the discussion than anything else.” In addition, instructor feedback was also valued by students. Students expressed that “your feedback has been thoughtful and helpful.” The value of instructor feedback was supported by the end-of-course evaluation items: “The instructor provided feedback on my performance within the time frame noted in the syllabus.” (M = 4.9) and “The instructor's feedback on my work was helpful” (M = 4.6).

As to the paper analysis activity, students appreciated it as an opportunity to apply academic skills and to develop deeper understandings. For the paper analysis assignments, students read assigned research papers each week and critically analyzed the advantages and disadvantages of the papers regarding the research purpose, research design, data collection, data analysis, findings, conclusions, and implications. As a student noted in the course evaluation, “the thoughtfulness of the paper analysis is helpful to understanding topics at a deeper level.” Shawn also highlighted it in the interview:

*I got the most benefit out of the paper analysis. Man, I did not like doing those. But I will say for the first time ever in all of my classes I actually felt like I was doing academic work, where I actually kind of felt like a researcher. I thought I actually used some skills that I never really used before*.

#### Ineffective strategies

**Fig. 2:**
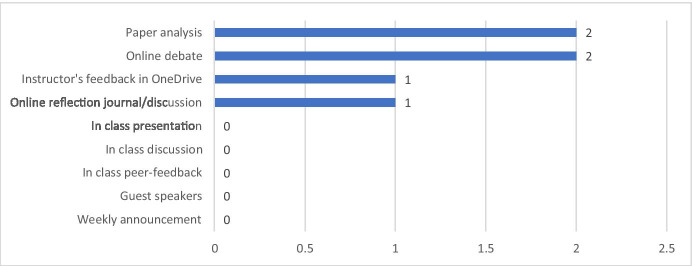
Learning activities that students perceived as ineffective

Some students identified the online debates, online discussions, and paper analyses as ineffective strategies (see Fig. 2). Students thought that the online debate was ineffective mostly because the rules of the activity limited them in expressing their own thoughts and ideas. In the interview, Lana voiced, “I felt like the online debates with the other school could have been really cool. But they just fell flat because we weren't really debating. We were repeating what had been given to us and basically just summarizing.” Moreover, Cindy said: “I would have preferred a more, you know, an in-person debate, I thought the debate maybe was a misnomer because it wasn't a back-and-forth where we were trying to arrive at a consensus.” In the course evaluation, a student commented, “it was more like a reflection on an article than a debate. It was a great experience collaborating with students from a different university.”

The analysis of students’ posts in the online debate supported the above claims. Students’ debate was very organized within the group. Students in each group posted six posts in total. However, this organization limited the flexibility of expressing students’ personal thoughts. The instructor reflected on the online debate assignment and noticed that the online debate activities were not well-designed. The online debate should be generated from students’ personal perspectives after the reading, rather than a simple summary of the assigned reading materials. In addition, coordinating among students from two institutions in the asynchronous debate was challenging. If the students from the other university could not finish their part on time, they disrupted the work of the students who were debating with them.

Students’ perceptions of the ineffective online reflection journals/discussions were manifested in their weekly discussions and interviews. Student’s participation in the online reflective journals/discussions decreased as time went by (see Table 2). In the first week of the reflective journals/discussions, besides posting their original journals in the discussion forum, students commented on each other’s posts and interacted with peers. However, students were not required to comment on each other’s posts. As the course proceeded, the number of posts and words of posts decreased through four weeks, as indicated in Table 2. In the interview, Sam explained why he thought it was ineffective:

*I didn't feel like I got a lot out of online discussions. I felt that they were better than nothing in terms of learning the material. But they didn't give me the structure that I needed to reflect thoroughly on the reading that we had done*.

**Table 2:** Weekly online journal reflection/discussion

Week	Number of posts	Total Number of words
Week 2	28	5754
Week 9	21	4087
Week 10	7	2841
Week 12	9	3438

The paper analysis was considered the most difficult assignment in this course. Two students disliked it and complained that it was difficult and time-consuming. As a student mentioned in the course evaluation, “I actually value the activity; it just takes way too long! I've taken many courses in my life, and this activity has taken the most toll on my emotions.” The instructor noted that this course was the first research-focused course for the graduate students, and thus it would have been better to introduce such a demanding activity with more scaffolding efforts.

Students didn’t like the use of OneDrive for feedback due to the additional cognitive load the technology imposed. They would prefer to minimizing the number of technologies required to succeed in a single course. One student mentioned that “This adds a layer of complexity that is not necessary. It is possible to provide the feedback you give within Canvas.”

### RQ2 From graduate students’ perspective, what are the advantages and disadvantages of BL?

#### Advantages of blended learning

Participants identified a few advantages of BL, such as learning community, interactions, and immediate feedback. Students valued the learning community they were building tighter in this blended course. Zoe stated that “blended classes create a sense of belonging and give an opportunity to bounce ideas off one another.” Similarly, Lana elaborated that:

*Online courses make me feel disconnected from my classmates, whereas with a blended learning situation, you meet occasionally. You get to see people… I felt like in the blended learning environment, I had a chance to get to know my classmates and their strengths.* (interviewee transcript A, p2, line 91-94)

In addition, interactions and immediate feedback were other advantages valued by students. In the interview, Sam said, “I think that timeliness of lessons is an advantage in a blended solution, especially if you're going to use synchronous webinars as a part of that blended solution.” Shawn expressed similar thoughts below:

*Well, the advantages are the human interaction. The fact that you know when you have an idea or a thought, and you can bounce it off somebody, and they give you feedback within five seconds. It means so much more than if you type up some big paper, you submit it, and then you hear back four or five days later* (interviewee transcript B, p3, line166-174)

#### Disadvantages of blended learning

Participants consistently pointed out a few disadvantages of BL, including both making efforts to attend face-to-face sessions and the various challenges in online learning. Lana expressed the challenges of physically showing up on campus below:

*I'd say I'm really fortunate that I have the GRA position as my primary gig. But that's not the norm for most people. For me showing up on campus isn't a big deal. I know for other students, it can be hard to attend classes.* (interviewee transcript A, p3, line138-143)

On the contrary, other disadvantages were related to individual learning in online sessions. Sam said: “learners are not physically together [in online sessions]. And by that, I mean adult learners oftentimes learn best from others.” Shawn expressed the motivation perspective of learning on their own “if you're not very motivated intrinsically to just do some of the work on your own, that can be difficult.”

### RQ3 How can BL leverage learning technologies?

The variety of data generated a plethora of suggestions, but of which focused on simplicity and minimalism and offering support. The instructor of the course adopted Canvas, OneDrive, email, and Nuclino for content delivery and communications. Participants stressed that using the least amount of technologies possible is a key to a successful learning experience. Having more than one learning management system only adds complications instead of support. In the interview, Shawn said, “It is important to keep just one mode of communication, for example, Canvas, where all the instructions are provided.” To support students’ learning with technology, instructions are necessary on how to use the technologies deployed in the course at the beginning of the semester. As Zoe said, “You can't have the assumption that everyone knows how to use the technology. There needs to be a little bit more of instruction or simplification [of technology].” Similarly, Cindy stated, “I think it's best if the instructor gives some kind of instructions at the beginning of the semester like how to navigate those technologies and how to use them. I think that would be helpful.” In addition, students suggested that technology use should depend on what is available to students. Linda said:

*A lot of our students are rural. They don't have high-speed Internet. They can't do Zoom or WebEx because they just don't have a good width. Yeah, a lot of them still don't have computers at home… It's like you may not be able to use technology. It's gonna really depend on the resources your students have*. (interviewee transcript C, p5, line275-283)

## Discussion

A few instructional strategies were found effective in this study, including class discussions, presentations, peer-feedback, and paper analyses. Some of the asynchronous learning activities were less effective, such as online debates and discussions. Students appreciated BL as it fostered community building, increased interactions and interactivities, and empowered them with immediate feedback. However, F2F and online sessions may be challenging in various ways. For example, it was sometimes uneasy to commute to campus for F2F sessions, while students also needed more guidance on how to succeed in online learning. Students would also like to have simplified and streamlined technology applications in BL, and perhaps more importantly, with sufficient training and timely technical support.

### Effective strategies in BL

Interactions are critical in both F2F and online sessions in blended courses. This study found that F2F interactions in class, such as discussions and presentations, were effective in BL. Students reported that discussions in class helped them construct knowledge through active communications and dialogues from diverse perspectives, which was consistent with previous research (i.e., Hamann et al., 2012; Huerta, 2007; Vonderwell, 2003). In addition, interactions with peers also strengthened the sense of belonging and community, as supported by previous research as well (e.g., Bernard et al., 2009; Diep et al., 2017; Lidstone & Shield, 2010). Despite the various potential benefits of online discussions (Putman et al., 2012; Williams & Lahman, 2011), students considered asynchronous online discussions ineffective in BL. Specifically, the online debate was not successful in this study, partially due to flaws in the design of the activity. Therefore, appropriate design and facilitations of such interactive learning activities are critical in BL (Butz et al., 2014; Szeto & Cheng, 2016).

Immediate feedback was praised as effective in this study, confirming similar findings from prior studies (Aghaee & Keller, 2016; Fluckiger et al., 2010). As students provided formative feedback to peers in F2F classes, they were overall satisfied with instant feedback and the enriched learning experiences. Prior resources also acknowledged that immediate feedback could motivate learners (Denton et al., 2008) and increase learners’ satisfaction (Lee et al., 2011).

### Technology use in BL

Technology use is critical in BL. Participants in this study were not comfortable with the different technologies utilized in BL, even though they were all advanced graduate students in a learning design and technology program. They suggested simplifying and streamlining the use of technology and would request more technical support in BL. Learning technology can support BL in both F2F and online environments (Norberg et al., 2011). However, technology applications in BL should carefully address issues like learner’s preferences, access to technology, and students’ technical competencies. Despite the wide range of available learning technologies, BL should carefully limit students’ cognitive load by simplifying and supporting technology usage (Holley & Oliver, 2010; Johnson, 2017; Song et al., 2004).

### Implications for instructors and students in BL

Instructors should use strategies to increase the interaction among learners and build a learning community in blended courses. In the face-to-face class session, instructors could build a learning community by encouraging students to present their class projects, provide feedback to peers and discuss open-ended issues related to the course topics. In addition, instructors could leverage asynchronous discussions for students’ knowledge construction. However, instructors should provide scaffold and guidance to engage learners in asynchronous discussions.

Regarding technology use in BL, given that technical support can improve students’ satisfaction with the course (Lee et al., 2011), instructors should provide appropriate technical support. For example, instructors could create tutorial videos and instructions on using the technology in blended courses. Moreover, instructors should consider students’ learning experience regarding the technology in BL by constantly getting feedback from students.

Students in BL should familiarize themselves with the course learning objectives and the purposes and descriptions of each learning activity. Understanding the rationales behind the course design can help them navigate through the course. In addition, students could consider themselves as active knowledge constructors rather than passive information receivers. Being active learners and taking responsibility for their own learning can help them leverage the resources provided in both face-to-face and online sessions in BL.

## Limitations and suggestions for future research

A few limitations are noteworthy in this study. First, the study context was limited to a small-sized graduate class in learning design and technology, and thus the findings may not be applicable in other disciplines or at other educational levels. Future research could expand the study in diverse educational settings. Second, the number of interviewees was limited. Thus, instructors and instructional designers should be cautious when applying the findings of this study to larger classes. Third, the interviewer and interviewees were peers with a strong rapport, which must have influenced the research in various ways. To establish trustworthiness, the researchers worked collaboratively to address possible biases and conducted interviews two months after the course had concluded. Fourth, even though the research involved multiple data sources, it did not examine students’ learning outcomes. Future studies could investigate more closely the effect of strategies and technologies on students learning outcomes in BL.

Due to the pandemic, BL will most likely continue and increase in varied forms. This study is therefore particularly meaningful for instructors and instructional designers. Instructors and instructional designers should encourage interactions among learners, provide immediate feedback, leverage peer feedback, and simplify technology usage, and facilitate technology use in blended courses.

## Data Availability

The datasets used and/or analyzed during the current study are not publicly available due to their personal and private nature but are available from the corresponding author on reasonable request.
